# Documenting the Human Health Impacts of Climate Change in Tropical and Subtropical Regions

**DOI:** 10.4269/ajtmh.16-0400

**Published:** 2016-08-03

**Authors:** Jennifer M. Kreslake, Mona Sarfaty, Edward W. Maibach

**Affiliations:** ^1^ Center for Climate Change Communication, George Mason University, Fairfax, Virginia.

Climate change is harming human health, and the magnitude of the harm is increasing.^1^ This is especially true in tropical and subtropical regions that are vulnerable to greater intensity, frequency, and duration of extreme weather, such as hurricanes, drought, and increases in heat, as a result of climate change.^2^ Nearly all countries situated in the geographic tropics are poor, and therefore have fewer resources to adapt to impacts of climate change.^3^,^4^ Protecting the public's health in these regions from serious—potentially catastrophic—harm associated with climate change requires coordinated response from tropical medicine and global health professionals, and from leaders of civil society more broadly.

## Implications of Climate Change for Tropical Medicine

For developing countries in tropical and subtropical regions, infrastructure challenges combine with environmental conditions to increase health-related vulnerabilities to climate change. Industrial and vehicle emissions in hot, humid cities contribute to poor air quality due to smog, resulting in increased morbidity and mortality from respiratory diseases.^5^,^6^ Acute health effects of storms and flooding include injury and death, as well as indirect effects such as compromised sanitation systems that contribute to increased incidence of diarrheal diseases.^7^–^10^ Heat, drought, and extreme weather events impact agricultural production and threaten food security, particularly for populations that rely on subsistence farming.^8^ Warmer, humid conditions will expand habitats for insect and zoonotic disease vectors and contribute to accelerated vector breeding and pathogen maturation and multiplication within the vector (e.g., as with malaria).^11^ Beyond regional health outcomes, the preponderance of climate change impacts in tropical and subtropical regions has implications for global health security, with population displacement and migration from environmental changes playing a role in conflict, economic challenges, and social upheaval, as well as emerging infectious disease transmission.^12^–^14^

## Global Governance and Local Responses to Climate Change

Climate change will require systematic and sustained disaster risk management. In addition to relief efforts, public health responses include prevention and preparedness before disasters occur.^15^ National and international guidelines should inform local planning activities.

Climate change adaptation (preparedness) and mitigation (prevention) can occur through policies and interventions at international, national, and local levels. In December 2015, 176 countries and the European Union signed the Paris Agreement, a treaty under the United Nations Framework Convention on Climate Change committed to the reduction of greenhouse gas emissions, enhanced adaptation efforts, and alignment of financial activities with climate-resilient development goals.^16^,^17^ This treaty begins to address the driving forces and pressures contributing to climate change that can be managed through national policies in signatory countries, such as those concerning greenhouse gas emissions, energy, transport, agriculture, and land use.

In support of the Paris Agreement, the World Health Organization (WHO) encourages strengthening health system capacity and documentation of vulnerabilities and policy responses to climate change.^18^,^19^ The WHO provides guidance for national or local preparedness for the health impacts of climate change, including vulnerability, impact, and adaptation assessments.^20^

## Risk Communication to Regions Experiencing Acute Health Impacts of Climate Change

Public perceptions of disaster risk are influenced by previous personal experiences with the natural hazard and levels of trust in authorities and experts.^21^ Failed preparedness and response efforts can damage trust in authorities, rendering individuals less likely to comply with recommendations from these sources in future hazards.^22^ Thus, sustained, well-planned, and culturally relevant public health responses to climate change impacts are paramount. Maintaining accurate estimations of risk is important (i.e., not overinflating personal risk from hazards, yet providing necessary information) to sustain public trust and engagement.^23^ For recurring hazards, risk communication should activate individuals' recall of previous experiences, or indirect experience from witnessing media reports of hazards, when encouraging protective actions. For novel or emerging environmental risks, authorities should provide clear recommendations based on what is known, be transparent about areas of uncertainty, and maintain ongoing communication as information about appropriate protective action becomes available.^21^,^24^

## Climate-Sensitive Injury and Disease Surveillance

In addition to tracking extreme weather events leading to hazards, injury and disease surveillance is needed to help public health officials respond efficiently to climate-sensitive health impacts and improve local clinical capacity. Indicators of these health impacts must be relevant to vulnerable populations and/or tropical and subtropical regions. Indicators that have been proposed in the United States^25^ and Canada^26^ include environmental conditions, morbidity and mortality from injury and disease, vulnerability (e.g., proximity to hazards), mitigation and adaptation efforts, and the policy environment. These are not exhaustive and may require further refinement to be applicable to tropical and subtropical regions.

To properly prepare for the health burdens presented by climate change, public health agencies, health professionals, and researchers must document changes occurring in climate-sensitive health outcomes in tropical and subtropical regions. In addition, descriptions of the status of infrastructure and clinical capacity to respond to these impacts are needed, particularly among developing countries.

## Assessing Health Vulnerabilities in the Solomon Islands

In a striking early example of the observed effects of climate change, recent research has demonstrated that the Solomon Islands are disappearing due to shoreline change as a result of global sea-level rise, destroying villages and leading to community relocation.^27^ In this issue of the *American Journal of Tropical Medicine and Hygiene*, Natuzzi and others detail the human costs associated with this phenomenon.^28^

The Natuzzi study provides the necessary information for localities to plan for the health implications of climate change they are currently experiencing, and will increasingly face in the future. The authors analyze infrastructure challenges and human health vulnerabilities in a Solomon Island city during extreme downpours and subsequent flooding. The study uses a variety of surveillance sources to identify multiple direct impacts of this extreme weather event on public health: acute morbidity and mortality (e.g., blunt force trauma and drowning), subacute infectious disease impacts, influenza-like illness, vector-borne illness, and diarrhea. The authors use a geographic information system (GIS) to map population proximity to flooding risk and health system capacity, linking disparate sources such as census estimates, hospital bed data, and evaluation data from healthcare site visits. GIS technology is well suited to studies of climate change and health, given the close relationship between geographic features and population-level health outcomes. Strengthened surveillance systems and ongoing refinement of GIS analytic techniques will benefit climate change adaptation and mitigation efforts.

The events in the Solomon Islands are not expected to be an isolated occurrence, and climate change will result in epidemiological changes throughout the tropics. Physicians in tropical and subtropical regions (including Ecuador, Brazil, Argentina, Chile, Peru, Turkey, Cyprus, Georgia, Nigeria, Oman, Egypt, Saudi Arabia, Indonesia, Bangladesh, India, the Philippines, China, Australia, and French Polynesia) have observed patient health outcomes they attribute to climate change.^29^ Systematic reporting of such outcomes in regionally specific contexts is urgently needed.

## Conclusions

The field of tropical medicine will confront some of the first, most widespread, and most pronounced human health impacts of climate change. Documenting these health outcomes in localized contexts will enable practitioners to target treatment according to health vulnerabilities, highlight regional adaptation needs for public health agencies, and establish evidence of the earliest and mounting health consequences of climate change for policymakers and the public. These data are powerful tools for climate change adaptation and mitigation. Key public health principles, including prevention and preparedness, risk communication, and surveillance must be mobilized in the context of climate change. Individuals who, by virtue of geography or socioeconomic position, are most vulnerable to the acute consequences of climate change urgently require protection. More broadly, these efforts are crucial to global health interests, and serve as a harbinger of the integral role that the health sector will occupy in climate change adaptation and mitigation.
